# Interferon regulatory factor 8/interferon consensus sequence binding protein is a critical transcription factor for the physiological phenotype of microglia

**DOI:** 10.1186/1742-2094-9-227

**Published:** 2012-09-28

**Authors:** Makoto Horiuchi, Kouji Wakayama, Aki Itoh, Kumi Kawai, David Pleasure, Keiko Ozato, Takayuki Itoh

**Affiliations:** 1 Department of Neurology, University of California Davis, School of Medicine, 4860 Y Street, Sacramento, CA, 95817, USA; 2 Institute for Pediatric Regenerative Medicine, 601A Shriners Hospitals for Children Northern California, 2425 Stockton Boulevard, Sacramento, CA, 95817, USA; 3 Department of Advanced Clinical Science and Therapeutics, Graduate School of Medicine, The University of Tokyo, 7-3-1 Hongo, Bunkyo-ku, Tokyo, 113-8655, Japan; 4 Department of Medical Technology, Graduate School of Medicine, Nagoya University, 1-1-20 Daikou-minami, Higashi-ku, Nagoya, 461-8673, Japan; 5 Laboratory of Molecular Growth Regulation, Program in Genomics of Differentiation, National Institute of Child Health and Human Development, National Institutes of Health, 6 Center Drive 2753, Bethesda, MD, 20892, USA

**Keywords:** Microglia, Interferon regulatory factor, Phagocytosis, Cytokine, Cuprizone-induced demyelination

## Abstract

**Background:**

Recent fate-mapping studies establish that microglia, the resident mononuclear phagocytes of the CNS, are distinct in origin from the bone marrow-derived myeloid lineage. Interferon regulatory factor 8 (IRF8, also known as interferon consensus sequence binding protein) plays essential roles in development and function of the bone marrow-derived myeloid lineage. However, little is known about its roles in microglia.

**Methods:**

The CNS tissues of IRF8-deficient mice were immunohistochemically analyzed. Pure microglia isolated from wild-type and IRF8-deficient mice were studied *in vitro* by proliferation, immunocytochemical and phagocytosis assays. Microglial response *in vivo* was compared between wild-type and IRF8-deficient mice in the cuprizon-induced demyelination model.

**Results:**

Our analysis of IRF8-deficient mice revealed that, in contrast to compromised development of IRF8-deficient bone marrow myeloid lineage cells, development and colonization of microglia are not obviously affected by loss of IRF8. However, IRF8-deficient microglia demonstrate several defective phenotypes. *In vivo*, IRF8-deficient microglia have fewer elaborated processes with reduced expression of IBA1/AIF1 compared with wild-type microglia, suggesting a defective phenotype. IRF8-deficient microglia are significantly less proliferative in mixed glial cultures than wild-type microglia. Unlike IRF8-deficient bone marrow myeloid progenitors, exogenous macrophage colony stimulating factor (colony stimulating factor 1) (M-CSF (CSF1)) restores their proliferation in mixed glial cultures. In addition, IRF8-deficient microglia exhibit an exaggerated growth response to exogenous granulocyte-macrophage colony stimulating factor (colony stimulating factor 2) (GM-CSF (CSF2)) in the presence of other glial cells. IRF8-deficient microglia also demonstrate altered cytokine expressions in response to interferon-gamma and lipopolysaccharide *in vitro*. Moreover, the maximum phagocytic capacity of IRF8-deficient microglia is reduced, although their engulfment of zymosan particles is not overtly impaired. Defective scavenging activity of IRF8-deficient microglia was further confirmed *in vivo* in the cuprizone-induced demyelination model in mice.

**Conclusions:**

This study is the first to demonstrate the essential contribution of IRF8-mediated transcription to a broad range of microglial phenotype. Microglia are distinct from the bone marrow myeloid lineage with respect to their dependence on IRF8-mediated transcription.

## Introduction

Microglia, the resident mononuclear phagocyte (MP) system of the CNS, play principal roles in the innate immune response to tissue damage and invasion of foreign organisms, and also in initiation and modulation of the adaptive immune response [1-3]. Although microglia share many common features of MPs with bone marrow (BM)-derived monocyte lineage cells and tissue macrophages of monocyte origin, recent studies have demonstrated that the contribution of BM-derived myeloid cells to microglial homeostasis in the steady state is a far less frequent event than was previously believed [4,5]. Only under some abnormal conditions such as tissue alteration following irradiation to the CNS, do a subset of circulating monocytes (Ly-6C/Gr-1^hi^ CCR2^+^) become engrafted as BM-derived microglia (BMDM) through the CNS vasculature [5]. Moreover, *in vivo* fate mapping studies have established that microglia are an ontogenically distinct population in the MP system. Microglial precursors arise from primitive extra-embryonic hematopoiesis in the yolk sac prior to the onset of blood circulation. These primitive MPs migrate into the CNS through blood vessels independently of the second wave of hematopoiesis within the embryo proper, which leads to the adult (definitive) hematopoiesis in the BM [6]. Once having colonized the CNS, embryonic microglia are highly proliferative and strongly dependent on colony stimulating factor 1 receptor (CSF1R)-mediated signaling for development during embryogenesis. In mice lacking CSF1R, the number of microglia is greatly reduced [6,7], whereas circulating monocytes are present, indicating that, in contrast to microglia, the BM-derived monocytes are less dependent on CSF1R-mediated signaling for their development [6]. These accumulating pieces of evidence further support the notion that microglia are maintained by self-renewal as a distinct MP population without continuous replenishment by systemic BM-derived precursors or myeloid cells, although it remains to be clarified to what extent microglia share common molecular mechanisms with BM-derived MPs in their development, maintenance, and functioning.

The molecular basis of myeloid development in the BM hematopoiesis has been intensively studied. Among the molecules known to be involved in myeloid development to date, interferon regulatory factor-8 (IRF8, also known as interferon consensus sequence binding protein (ICSBP)), a member of the interferon regulatory factor family, acts as an essential transcription factor for differentiation and maturation of BM-derived MPs in the myeloid lineage as well as for B cell development from hematopoietic stem cells [8-10]. IRF8 limits the size of the bipotential granulocyte-macrophage progenitor pool, and directs these progenitor cells to differentiate into the monocyte lineage by stimulating expression of genes critical for MP differentiation and by repressing a series of genes required for granulocytic differentiation [11]. IRF8-deficient BM-derived MPs are not generated efficiently in IRF8-deficient mice, and are also defective in production of cytokines and reactive oxygen species [12-14]. IRF8 is constitutively expressed in microglia as well [15]. Since microglia arise from mesodermal myeloid progenitors in the primitive hematopoiesis, which is distinct from the development of BM-derived MPs, it is essential to determine how IRF8 regulates development of microglia to understand the molecular basis of distinct development of microglia and BM-derived MPs. Moreover, given the essential roles for IRF8 in the functional phenotypes of BM-derived MPs, constitutive expression of IRF8 could also be critical for microglial functions. Indeed, a recent study has demonstrated an essential role for IRF8 in activation of microglia in the spinal cord following peripheral nerve injury [16].

In this study, we performed a comprehensive study of the microglia of constitutional IRF8-null mice, and further demonstrate essential roles for IRF8 in physiological phenotype and function of microglia.

## Materials and methods

### Animals

The mouse strain with targeted null mutation in the *Irf8* gene in this study was reported previously [17]. The strain was backcrossed onto the C57BL/6J strain for more than 5 generations. Animals were housed in standard laboratory cages with unrestricted access to food and water, and maintained under 12 h light/dark cycles. All procedures using the animals were approved by the Institutional Animal Care and Use Committee of the University of California, Davis.

### Reagents and chemicals

All reagents and culture media used in this study were purchased from SIGMA (St. Louis, MO, USA) and Invitrogen (Carlsbad, CA, USA), respectively, except for the following products. Mouse anti-β-actin antibody was from Cell Signaling Technology (Danvers, MA, USA). Rabbit anti-IBA1/AIF1 antibody was from Wako Chemicals USA (Richmond, VA, USA). Rabbit anti-IRF8 antibody was reported previously [18].

### Cell culture

#### Mixed glial culture

Whole brains from 0 to 2-day-old mice were dissected and diced into 2-mm cubes. After cleaning off meninges, and vessels including choroidal plexus, the brain chunks were digested by 20 units/ml papain in Earle’s buffered salt solution supplemented with 1 mM L-cystaine, 0.5 mM EDTA, 0.36% (w/v) D-glucose, and 250 units/ml DNase at 33°C for 90 min. Papain was inactivated by 1.5 mg/ml ovomucoid in PBS containing 1.5 mg/ml bovine serum albumin (BSA) and 250 units/ml DNase. The softened chunks were gently triturated by passing through a 1-ml serological pipette several times. The resulting suspension was left for 1 minute to allow undissociated tissues to settle down on the bottom, and then the supernatant containing dissociated cells was collected to another tube. After repeating this trituration step three times, the obtained cell suspension was spun at 220 × g for 15 minutes. Cells were resuspended in the MG medium; 5% (v/v) heat-inactivated fetal bovine serum, 1× N2 NeuroPlex supplement (Gemini Bio-Products, West Sacramento, CA), 100 units/ml penicillin, and 100 μg/ml streptomycin in high glucose Dulbecco’s modified Eagle’s medium (DMEM), and plated on a poly-D-lysine-coated T75 tissue culture flask. In some experiments, 20 ng/ml of either macrophage colony stimulating factor (M-CSF) or granulocyte-macrophage colony stimulating factor (GM-CSF) was added to the culture medium at 5 days *in vitro*, by which time most cultures had reached confluency. Cultures were fed with fresh medium every other day.

#### Magnetic cell sorting (MACS) to isolate CD11b^+^ mouse microglia

Mixed glial cells in a T75 flask were maintained in the presence of 20 ng/ml M-CSF from day 5 to day 9 *in vitro*. Then cells were washed with 5 ml Ca^++^- and Mg^++^-free Hanks’ balanced salt solution (HBSS-) and then 1 ml trypsin-EDTA (0.05% (v/v) and 0.53 mM, respectively, in HBSS-) was added. After a 3 minute-trypsinization at 37°C, 0.5 ml of trypsin inhibitor-BSA-DNase (0.25 mg/ml, 1.44 mg/ml and 60 units/ml, respectively, in DMEM.) was added, and detached cells were collected into a 50-ml tube containing 3.5 ml MG medium in addition to the medium saved from the cultures and from the HBSS- that had been used for wash. Cells were collected by centrifugation at 520 × g for 5 minutes, resuspended into a mixture of 40 μl CD11b microbeads (catalog number: 130-093-634, Miltenyi Biotech, Auburn, CA, USA) and 160 μl ice-cold MACS buffer (0.5% BSA, 2 mM EDTA in PBS, pH 7.2), and incubated at 4°C for 15 minutes. After a wash with 20 ml ice-cold MACS buffer, cells were resuspended into 500 μl ice-cold MACS buffer, and then applied to a MACS separation column LS (Miltenyi Biotech) in a magnetic field. The column was washed with 3 ml ice-cold MACS buffer three times and removed from the magnetic field. Magnetic labeled cells were eluted with 5 ml MACS buffer. After centrifugation at 520 × g for 5 minutes, cells were plated on to poly-D-lysine-coated culture ware at 1.25 × 10^3^ cells/cm^2^ in MG medium.

### Immunocytochemistry

Purified microglia were incubated with rat anti-CD11b antibody (1:50; AdB Serotec, Raleigh, NC, USA) for 30 minutes. After washing with PBS three times, cells were incubated with Alexa Fluor™ 488-conjugated goat anti-rat IgG for 30 minutes. Cells were fixed with 4% paraformaldehyde at room temperature for 15 minutes, and then permeabilized with ice-cold 100% methanol for 20 minutes. Nuclei were counterstained with 4,6-diamidio-2-phenylindole (0.5 μg/ml, DAPI).

### Immunoblots

Protein lysates were prepared in the lysis buffer as described previously [19]. Twenty μg of protein from each sample were size-fractioned by SDS-polyacrylamide gel electrophoresis, transferred onto a nitrocellulose membrane (Schleicher > Schnell, Keene, NH, USA) and probed with primary antibodies for IRF8 (1:5000) and AIF1/IBA1 (1:5000) for 1 h. Full range recombinant Rainbow Molecular Weight Markers (Amersham Biosciences, Piscataway, NJ, USA) were used as a reference for molecular sizes. Immunoreactive signals were detected by enhanced chemiluminescence according to the manufacture’s protocol (Amersham Biosciences). Equal protein loading was confirmed by subsequent probing with the mouse monoclonal antibody against β-actin (1:1000) in each experiment. 

### Flow cytometry

Mixed glial cells were collected by trypsinization and stained with anti-CD45 and anti-CD11b antibodies together with anti-F4/80, anti-CD11c, or anti-Ly-6G antibody for 30 minutes at room temperature. Each antibody was diluted at 1:100 in PBS containing 1% (w/v) BSA and Fc-blocker (1:40; BD Bioscience). Samples were acquired by Cyan-ADP flow cytometry (DakoCytomation, Carpinteria, CA, USA) and analyzed with Summit software (DakoCytomation). Antibodies used for flow cytometry are listed in Table 1.

**Table 1:** List of the fluorochrome-labeled monoclonal antibodies used for flow cytometry

**Antigen**	**Fluorochrome**	**Clone**	**Isotype**	**Company**
**CD11b**	FITC, PE	M1/70	Rat IgG2b, κ	BD Biosciences
**CD45**	eFluor450	30-F11	Rat IgG2b, κ	eBioscience
**F4/80**	PE	BM8	Rat IgG2a, κ	eBioscience
**CD11c**	APC	HL3	Ar Ham IgG1, λ2	BD Biosciences
**Ly6-G**	FITC	1A8	Rat IgG2a, κ	BD Biosciences

BD Biosciences (San Jose, CA, USA); eBioscience (San Diego, CA, USA).

### EdU (5-ethynyl-2′-deoxyuridine)-incorporation assay

Mixed glial cultures were incubated with 10 μM EdU for 6 h. Then, trypsinized cells were collected and stained with FITC-conjugated rat anti-CD11b antibody (1:100; BD Bioscience) in PBS containing 1% (w/v) BSA and Fc-blocker (1:40) for 30 minutes at room temperature. After washing with PBS containing 1% (w/v) BSA three times, cells were fixed with 4% (w/v) paraformaldehyde and permeabilized with saponin-based permeabilizing buffer. EdU was detected using a pacific blue-conjugated azide (Invitrogen) according to the manufacturer’s instruction. Samples were acquired by Cyan-ADP flow cytometry, and the number of EdU-positive cells in either CD11b-positive or negative cell population was separately counted by gating with Summit software.

For purified microglial cultures, EdU (10 μM as a final concentration) was added to pure microglia plated on poly-D-lysine-coated 12 mm-diameter round cover slips at 5×10^4^ cells/cover slip at 18 h after addition of MG alone, or MG medium supplemented with 20 ng/ml M-CSF or GM-CSF. After incubation for 6 h, cells were fixed and EdU incorporated into the nucleus was detected with azide conjugated with Alexa Fluor™ 594 according to the manufacturer’s instruction (Click-iT™ EdU cell proliferation assay, Invitrogen). All nuclei were counted with the aid of DAPI.

### Phagocytosis assay by flow cytometry

Microglia plated on a 24-well plate were incubated with Alexa Fluor™ 488-conjugated zymosan A particles (Invitrogen) at 2×10^6^ particles/ml in the MG medium. After wash with 0.5 ml HBSS- twice, cells were detached by trypsinization and collected into a 15-ml tube. After centrifugation at 520 × g for 5 minutes, cells were resuspended into 0.5 ml 0.1% (w/v) BSA in PBS, and then analyzed by CyAn-ADP flow cytometer. Mean fluorescent intensity (MFI) was calculated with Summit software.

### Real-time PCR

Real-time PCR (qPCR) analyses were performed by MX3005P (Stratagene, La Jolla, CA, USA) using TaqMan™ Assay-on-Demand™ assay kits (assay nos.: Mm01250092_g1, Mm01270606_m1, Mm00479862_g1, Mm00439546_s1 and Mm00434174_m1 for detection of IRF8, PU.1/SFPI1, AIF1/IBA1, IFNB1, and IL12B cDNA, respectively). For standardization, β-actin cDNA levels were also quantified with the kit (Mm00607939_s1), and the absolute cDNA amounts were expressed as ratios to β-actin cDNA. We analyzed each gene using total RNA samples from at least triplicated independent experiments.

### Immunohistochemistry

Mice were perfusion-fixed with 4% (w/v) paraformaldehyde in PBS, and tissues were processed as reported previously [20]. Tissue sections (6 μm-thick) were prepared by a cryostat (CM1950, Leica Microsystems, Watzlar, Germany); rinsed in PBS; incubated for 30 minutes at room temperature in a blocking solution containing 0.4% (w/w) Triton X-100, 10% (v/v) donkey serum, 15 mM HEPES, 0.02% (w/v) sodium azide in 1x minimum essential medium; and incubated with primary antibodies in the blocking solution overnight at 4°C. The following primary antibodies were used: rat monoclonal anti-CD11b (M1/70.15.11.5, 1:50; Developmental Studies Hybridoma Bank, Iowa City, IA, USA), rabbit polyclonal anti-PU.1/SFPI1 (#2266, 1:100; Cell Signaling, Danvers, MA), rabbit polyclonal anti-IBA1/AIF1 (No. 019-19741, 1:1000; Wako Chemicals USA, Richmond, VA, USA), rat monoclonal anti-F4/80 antigen (BM8, 1:100; eBioscience, San Diego, CA, USA) and rabbit polyclonal anti-CD68 (No. 250594, 1:200; AB Biotec, San Diego, CA, USA). Then, the sections were washed with PBS, incubated for 1 h in appropriate rhodamine, or Dylight 488-conjugated secondary antibodies (1:500, Jackson Immunoresearch, West Grove, PA, USA) at room temperature, and mounted with ProLong Gold antifade reagent containing DAPI (Invitrogen).

### Cuprizone-induced demyelination in mice

Demyelination was induced by feeding wild-type (*Irf8*^+/+^) and IRF8-deficient (*Irf8*^-/-^) male mice at 8 to 10 weeks of age a diet of pellet feed (Harlan Laboratories, Madison, MI, USA) containing 0.25% (w/w) cuprizone (Sigma) up to 6 weeks. Animals were sacrificed at the indicated time points for histological analyses.

### Myelin staining and Oil Red O staining

After fixation with paraformaldehyde as described above, brains were embedded in paraffin. Five μm-thick coronal brain sections between bregma -1.2mm and -2.2mm were stained with Luxol fast blue (LFB) (Sigma) for myelin together with periodic acid Schiff (PAS) for microglia and demyelinated axons. For Oil Red O staining, mice were perfusion fixed by 10% formalin. Brains were removed and postfixed, and then 6 μm-thick cryosections were prepared. NovaUltra™ Oil Red O Stain Kit (IHCWORLD, Woodstock, MD, USA) was used to stain undigested myelin lipids according to the manufacture’s instruction.

### Statistical analysis

Unless otherwise noted, data are presented as mean ± SD, and statistical significance was determined by two-tail ANOVA followed by Student-Newman-Keuls post hoc test. Results were considered significant when *P* < 0.05.

## Results

### IRF8-deficient microglia populated the CNS normally, but demonstrated less complex morphology with less IBA1 expression than wild-type microglia

**Figure 1:**
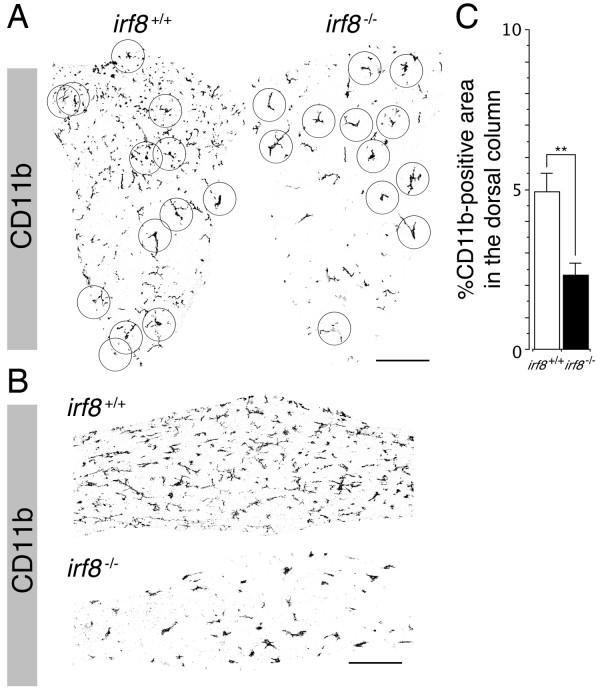
**IRF8-deficient microglia colonized the CNS normally, but could not be identified by AIF1/IBA1 immunoreactivity.** The spinal cords obtained from adult *Irf8*^+/+^ and *Irf8*^-/-^ mice were immunolabeled for various microglial markers (**A**), and positive cells in the dorsal column at the Th1 spinal level were quantified (**B**). Microglia were reliably identified as CD11b and PU.1 double positive cells in both *Irf8*^+/+^ and *Irf8*^-/-^ CNS tissues (pink arrows in far left panels in **A**). Note that few *Irf8*^-/-^ microglia demonstrated detectable immunoreactivity for AIF1/IBA1. Scale bar: 100 μm. **P < 0.01. (**C)** 20 μm-thick z-stack confocal images of the microglia immunolabeled for CD11b (green) and PU.1 (red) in the dorsal columns at the Th1 spinal level. CD11b-positive microglial processes were much sparser in the dorsal column of *Irf8*^-/-^ mice than those in *Irf8*^+/+^ mice, and processes of *Irf8*^-/-^ microglia were thicker and less elaborated than those of *Irf8*^+/+^ microglia. PU.1-positive microglial nuclei are indicated by arrows, demonstrating that similar numbers of microglia are distributed in the *Irf8*^+/+^ and *Irf8*^-/-^ spinal cords in contrast to the different density of the microglial processes. Scale bar: 100 μm.

Our immunohistochemical analysis of microglia in the CNS tissues of wild-type (*Irf8*^+/+^) and IRF8-deficient (*Irf8*^-/-^) adult mice first revealed that most *Irf8*^-/-^ microglia could not be identified as cells positive for AIF1/IBA1, a common marker protein for microglia. In contrast to AIF1/IBA1, *Irf8*^-/-^ microglia expressed other microglial markers such as CD11b, CD68 and F4/80 at levels comparable with those of *Irf8*^+/+^ microglia. Among these markers, we found that double immunolabeling for membrane CD11b and nuclear PU.1/SFPI1 (SPI1 in humans), a transcription factor exclusively expressed in hematopoietic lineage cells, is the most reliable method to identify microglia in both *Irf8*^+/+^ and *Irf8*^-/-^ CNS tissues particularly for quantitative analysis (Figure 1A). In the corpus callosum and the dorsal column of the spinal cord, nearly 100% of CD11b-positive microglia were positive for nuclear PU.1/SFPI1. Quantitative analysis of microglia in the dorsal column of the spinal cord using these microglial markers revealed that the proportion of microglia to total cells was not different between *Irf8*^-/-^ and *Irf8*^+/+^ mice (Figure 1B). These results indicate that, in contrast to compromised development of the BM myeloid lineage in *Irf8*^-/-^ mice, development and colonization of microglia are not obviously affected by loss of IRF8.

**Figure 2:**
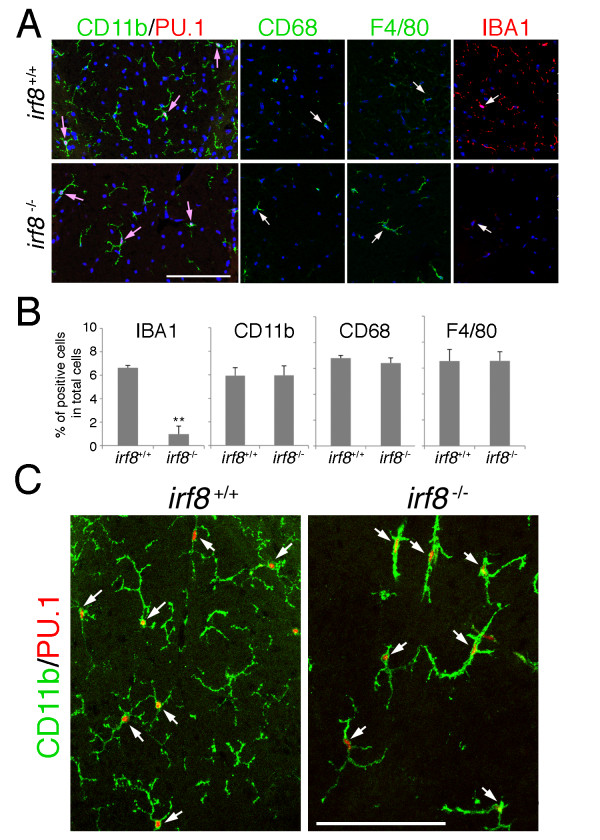
***Irf8***^**-/- **^**CNS tissues contain significantly less microglial processes than *Irf8***^**+/+ **^**tissues.** (**A)** Dorsal columns at the Th1 spinal level were immunolabeled for CD11b and PU.1, and inverted confocal fluorescence images of 12 μm-thick transverse sections are shown in monochrome. Only cross-sectional areas of the dorsal columns are shown. The location of each microglial nucleus positive for PU.1 is indicated by a circle to demonstrate similar distributions of microglia between the *Irf8*^+/+^ and *Irf8*^-/-^ dorsal columns, which contrasts to lower densities of CD11b-positive microglial processes in *Irf8*^-/-^ mice than in *Irf8*^+/+^ mice. (**B)** Similar difference was observed in the coronal sections of the corpus callosum. (**C)** The percentages of CD11b-positive areas in the whole cross-sectional area of the dorsal column were measured and compared quantitatively between *Irf8*^-/-^ (closed bar) and *Irf8*^+/+^ mice (open bar). Three 3-month-old animals were used in each genotype. **P < 0.01. Scale bar: 100 μm.

In addition to the reduced expression of AIF1/IBA1 in *Irf8*^-/-^ microglia, we also found a clear difference in their morphology *in vivo*. Generally, *Irf8*^+/+^ microglia in the normal CNS tissues extend elaborate and highly-branched processes which sometimes reach a distance of more than 50 μm from the cell soma and cover a broad area. As a good example, transverse sections of the *Irf8*^+/+^ dorsal column contain numerous CD11b-positive processes, some of which were from the microglia outside of the sections. In contrast, CD11b-positive *Irf8*^-/-^ microglial processes in the dorsal column were apparently sparser than those in the *Irf8*^+/+^ counterpart (Figure 1C, Figure 2). This difference was statistically significant when total CD11b-positive areas in the whole cross-sectional area of the dorsal column were compared quantitatively between *Irf8*^+/+^ and *Irf8*^-/-^ mice. We observed a similar difference in other CNS regions such as the corpus callosum (Figure 2). Confocal z-stack images demonstrated that the processes of *Irf8*^-/-^ microglia were generally thick and less extended compared with those of *Irf8*^+/+^ microglia (Figure 1C), suggesting that, since the density of microglia was similar between *Irf8*^+/+^ and *Irf8*^-/-^ mice, *Irf8*^-/-^ microglia were defective in forming normal extended processes with complex arborization.

### IRF8-deficient microglia did not proliferate well in mixed glial cultures, but demonstrated a hyperproliferative response to GM-CSF

**Figure 3:**
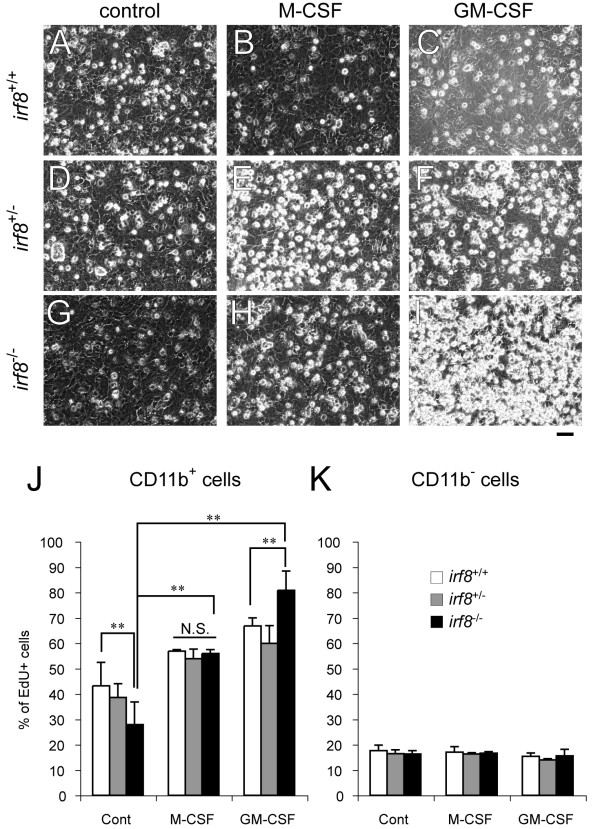
**IRF8-deficient microglia in the mixed glial cultures exhibited a hyperproliferative phenotype in response to M-CSF and GM-CSF. A**-**I**, Phase contrast images of mixed glial cultures from *Irf8*^+/+^ (**A**-**C**), *Irf8*^+/-^ (**D**-**F**), and *Irf8*^-/-^ (**G**-**I**) brain tissues. Cells were transferred to medium alone (control), medium supplemented with M-CSF (20 ng/ml) or GM-CSF (20 ng/ml) at 5 days *in vitro*, and then maintained in the same medium for another 4 days. Most microglia were seen as phase-bright round cells on the sheet of flat glial cells. Scale Bar: 50 μm. (**J**-**K)** Percentages of EdU-positive cells in total CD11b^+^ (**J**) or CD11b^-^ (**K**) cells in the mixed glial cultures of the respective genotypes. Cells treated medium alone (cont), or medium supplemented with M-CSF (20 ng/ml) or GM-CSF (20 ng/ml) for 4 days were exposed to EdU at 6 hours prior to fixation, and double-stained for CD11b and EdU. **P < 0.01 and N.S indicates no significant difference in a comparison of the two groups indicated.

We next studied *Irf8*^-/-^ microglia *in vitro*. We initially tried to isolate *Irf8*^-/-^ microglia by the standard ‘shaking-off’ method from the mixed glial cultures [21]. Compared with *Irf8*^+/+^ microglia, however, we found that *Irf8*^-/-^ microglia did not proliferate well in the mixed glial cultures, resulting in a much lower yield of pure *Irf8*^-/-^ microglia than that of *Irf8*^+/+^ microglia. Reduced proliferation rates of *Irf8*^-/-^ microglia in the mixed glial cultures were confirmed by EdU incorporation assay (Figure 3J), suggesting that *Irf8*^-/-^ microglia did not respond normally to undetermined mitotic signal(s) present in the mixed glial cultures. 

**Figure 4:**
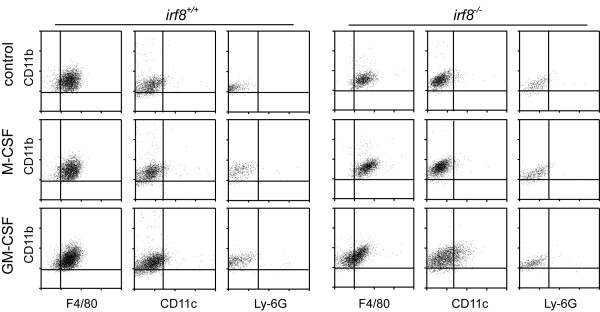
**F4/80, CD11c, and Ly-6G expression in *Irf8***^**+/+ **^**and *Irf8***^**-/- **^**microglia in the mixed glial cultures maintained in the presence of M-CSF or GM-CSF.** Mixed glial cells were cultured in medium alone (control) or medium supplemented with M-CSF (20 ng/ml) or GM-CSF (20 ng/ml) for 4 days, and labeled with anti-CD45 and anti-CD11b antibodies in combination with each of anti-F4/80, anti-CD11c or anti-Ly-6G antibodies. The bivariate plots show expression of CD11b, F4/80, CD11c and Ly-6G on gated CD45-positive cells. The quadrants were set using corresponding isotype controls.

Since *Irf8*^-/-^ BM myeloid progenitors are known to exhibit altered growth response to GM-CSF, G-CSF (granulocyte colony stimulating factor), and M-CSF, the cytokines involved in proliferation and differentiation of the myeloid lineage [22], we added M-CSF and GM-CSF to the *Irf8*^+/+^ and *Irf8*^-/-^ mixed glial cultures to examine the growth response of CD11b-positive microglia. Unlike *Irf8*^-/-^ BM myeloid progenitors, addition of M-CSF significantly enhanced proliferation of *Irf8*^-/-^ microglia in mixed glial cultures. Importantly, the rates of EdU incorporation were nearly equivalent between *Irf8*^+/+^ and *Irf8*^-/-^ microglia in the presence of M-CSF. In contrast, *Irf8*^-/-^ microglia in mixed glial cultures exhibited a hyperproliferative response to GM-CSF compared with *Irf8*^+/+^ microglia (Figure 3J). The lineage progression of *Irf8*^-/-^ BM myeloid progenitors is skewed toward cells of the granulocytic lineage even in the presence of M-CSF [22]. Therefore, we further examined alteration in the myeloid phenotype of *Irf8*^-/-^ microglia in response to either M-CSF or GM-CSF. Our results confirmed that M-CSF did not induce CD11c and Ly-6G, the dendritic cell (DC) and granulocyte markers, respectively, in both *Irf8*^+/+^ and *Irf8*^-/-^ microglia. On the other hand, GM-CSF reduced expression of F4/80 and increased CD11c-positive population in *Irf8*^-/-^ microglia (Figure 4). 

**Figure 5:**
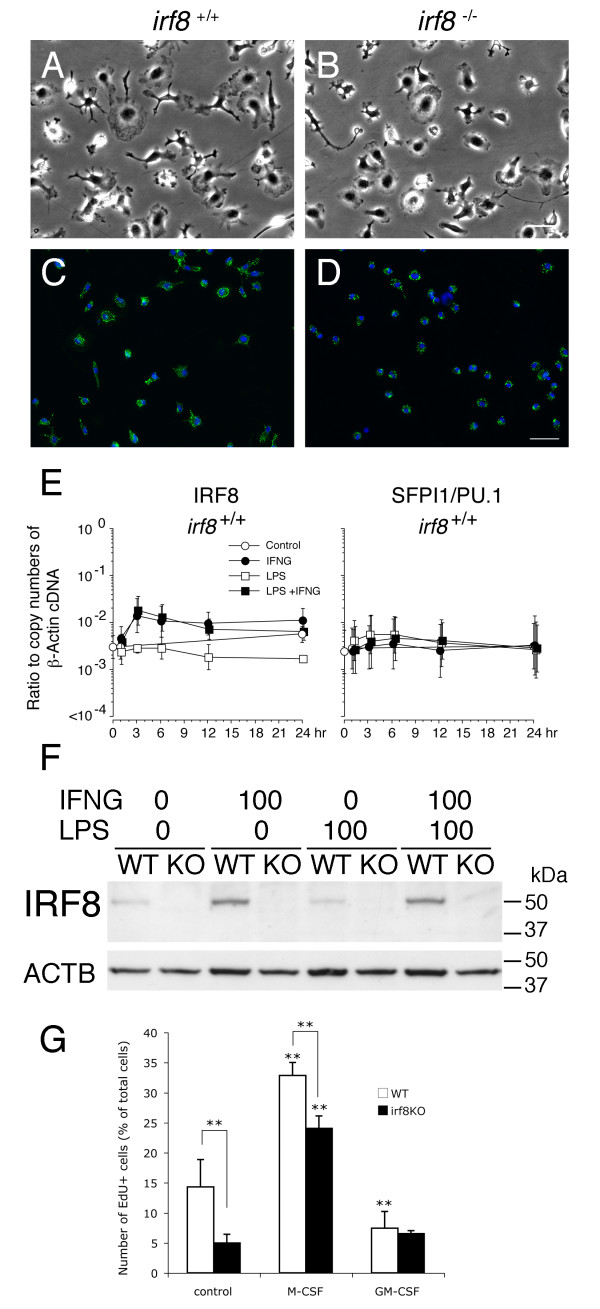
**Purified *Irf8***^**+/+ **^**and *Irf8***^**-/- **^**microglia *in vitro.*** (**A-D)** Representative phase contrast (**A**, **B**) and immunocytochemical (**C**, **D**) pictures of the microglia isolated from the *Irf8*^+/+^ (**A**, **C**) and *Irf8*^-/-^ (**B**, **D**) mixed glial cultures grown in the presence of M-CSF (20 ng/ml) by magnetic-activated cell sorting. Purified cells were immunolabeled for CD11b (green in **C**, **D**) at 24 h after isolation. Nuclei were counterstained with DAPI (blue). Scale Bar: 50 μm. (**E)***IRF8* and *PU.1/SFPI1* mRNA levels in the purified *Irf8*^+/+^ microglia. Purified microglia were incubated in medium alone (control) or in the presence of IFNγ (IFNG, 100 ng/ml), lipopolysaccharide (LPS, 100 ng/ml) or both for 24 h. Reverse transcribed *IRF8* and *PU.1/SFPI1* cDNA levels are plotted as ratios to copy numbers of β-actin cDNA on a logarithmic scale. (**F)** Immunoblots for IRF8 confirmed the results of mRNA. (**G)** Purified *Irf8*^-/-^ CD11b^+^ microglia were less proliferative than *Irf8*^+/+^ CD11b^+^ microglia even in the presence of exogenous M-CSF. Purified microglia were preincubated with medium alone for 24 h after isolation from *Irf8*^+/+^ (open bars) and *Irf8*^-/-^ (closed bars) mixed glial cultures, and then treated with medium alone (control), or medium supplemented with M-CSF (20 ng/ml) or GM-CSF (20 ng/ml) for 24 h. After a 6 hour incubation with EdU, EdU-positive and DAPI-positive nuclei were counted. **P < 0.01 in comparison with control or between the two groups indicated.

Based on these results, we isolated microglia from both *Irf8*^+/+^ and *Irf8*^-/-^ mixed glial cultures pretreated with M-CSF (20 ng/ml) for further *in vitro* analyses. To ensure the purity and maximum yield of microglia, the cells that were surface positive for CD11b were sorted out using MACS anti-CD11b microbeads. In the *Irf8*^+/+^ and *Irf8*^-/-^ microglial cultures which we obtained by this method, CD11b-positive cells were 96.2 ± 3.0% and 90.6 ± 2.3% of total cells, respectively, at 24 h after isolation. These *Irf8*^+/+^ and *Irf8*^-/-^ microglia were difficult to distinguish by morphology *in vitro* under a phase contrast microscope (Figure 5A-D). As reported by Campbell’s group [15], constitutive expression of *IRF8* mRNA and protein in the isolated microglia *in vitro* was confirmed by qPCR and immunoblotting, respectively. *IRF8* mRNA levels were comparable to those of *PU.1*/*SFPI1* mRNA in *Irf8*^+/+^ microglia, and further upregulated by interferon-γ but not by lipopolysaccharide (LPS). IRF8 protein levels were regulated in accordance with mRNA levels. No immunoreactivity for IRF8 was detectable in the isolated *Irf8*^-/-^ microglia (Figure 5E, F). Purified *Irf8*^-/-^ CD11b^+^ microglia demonstrated significantly lower EdU incorporation rates than *Irf8*^+/+^ CD11b^+^ microglia in control cultures and even in the presence of exogenous M-CSF. Interestingly, both *Irf8*^+/+^ and *Irf8*^-/-^ CD11b^+^ microglia no longer demonstrated proliferative responses to exogenous GM-CSF in the absence of concomitant other glial cells (Figure 5G). 

### IRF8-deficient microglia demonstrated a reduced maximum phagocytic capacity *in vitro*

Like their myeloid cousins, microglia are characterized by their motility and phagocytic activity. Motility of *Irf8*^+/+^ and *Irf8*^-/-^ microglia was analyzed *in vitro* by the same time-lapse video imaging as used in our previous study [23]. *In vitro* motility analysis failed to reveal significant differences between the two genotypes (data not shown).

**Figure 6:**
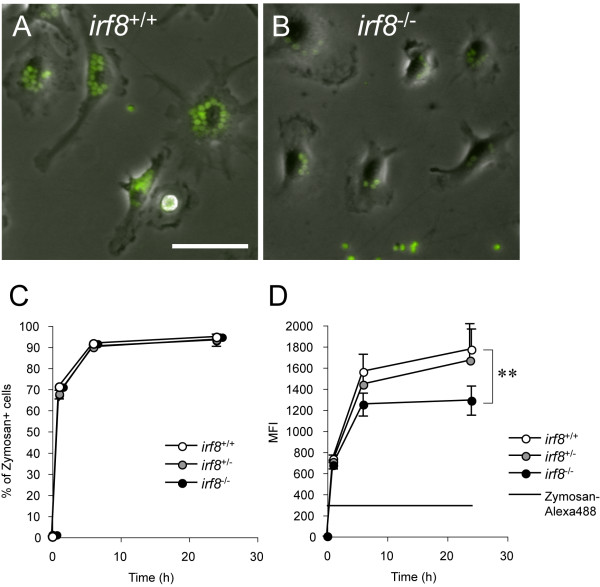
**IRF8-deficient microglia demonstrated a reduced phagocytic capacity *in vitro.*** Microglia from *Irf8*^+/+^ (**A**) and *Irf8*^-/-^ (**B**) mice were incubated with Alexa Fluor™ 488-conjugated zymosan particles for 24 hours. Scale bar: 50 μm. (**C-D)** Percentages of Alexa Fluor™ 488-positive cells in total cells (**C**) and mean fluorescent intensity (MFI) of Alexa Fluor™ 488-positive cells (**D**) were measured in *Irf8*^+/+^, *Irf8*^+/-^, and *Irf8*^-/-^ microglial cultures at 0, 1, 6, and 24 hour after adding the zymosan particles. The MFI of a non-phagocytosed Alexa Fluor™ 488-conjugated zymosan particle is also shown (Zymosan- Alexa488) in **D**. **P < 0.001 in comparison with the two groups indicated.

Alexa Fluor™ 488-conjugated zymosan particles were added to the pure microglial cultures to assess the phagocytic activity of *Irf8*^+/+^ and *Irf8*^-/-^ microglia by flow cytometry. Soon after addition of the particles, both *Irf8*^+/+^ and *Irf8*^-/-^ microglia started taking them up actively, and there was no difference in the percentages of the cells containing the fluorescent particles between *Irf8*^+/+^ and *Irf8*^-/-^ microglia throughout the experiment (Figure 6C), indicating that the molecular mechanism necessary for internalization of zymosan functioned normally in the absence of IRF8. On the other hand, we observed that *Irf8*^-/-^ microglia contained less zymosan particles than *Irf8*^+/+^ microglia when their phagocytic activities reached a plateau (compare Figures 6A and 6B). In support of this observation, the averaged fluorescence intensity was significantly reduced in *Irf8*^-/-^ microglia compared with *Irf8*^+/+^ microglia at 24 hr after addition of zymosan particles (Figure 6D). As the fluorescence emission or the profile of the excitation/emission spectra of the Alexa Fluor™ dye is unchanged over the pH range 4 to 9 [24], the averaged fluorescence intensity is quite likely to correlate with the amount of phagocytosed zymosan, thereby confirming that the maximum phagocytic capacity of *Irf8*^-/-^ microglia was reduced. 

### IRF8 was necessary to maintain the basal transcriptional level of AIF1/IBA1 in microglia, but not for its induction by interferon-γ

**Figure 7:**
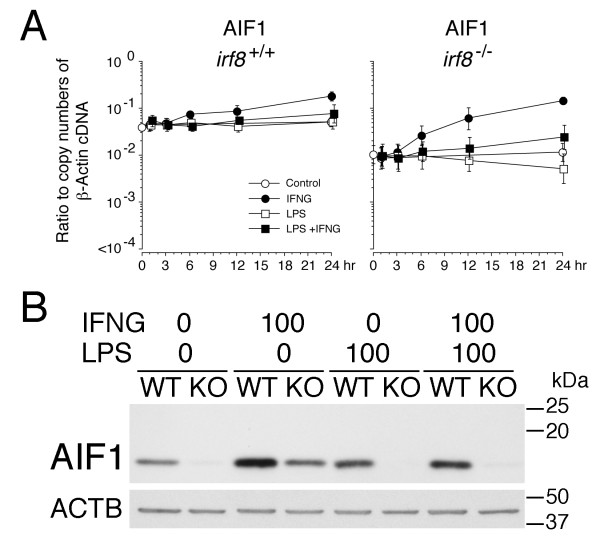
**AIF1/IBA1 expression in *Irf8***^**-/- **^**microglia was reduced but still inducible by IFNγ.** (**A)** Quantitative and kinetic analysis of *AIF1/IBA1* mRNA in *Irf8*^+/+^ and *Irf8*^-/-^ microglia after addition of medium alone (Control, open circle), IFNγ (IFNG, 100 ng/ml, closed circle), lipopolysaccharide (LPS, 100 ng/ml, open square) or both (closed square). Reverse transcribed cDNA levels at the indicated time points were quantified by qPCR and are plotted as ratios to copy numbers of β-actin cDNA on a logarithmic scale. Data were from at least three independent experiments. At time 0, data from controls are only shown. (**B) ***Irf8*^-/-^ microglia expressed far less AIF1/IBA1 protein than *Irf8*^+/+^ microglia. Purified microglia were maintained in the medium containing IFNγ (IFNG, 100 ng/ml), lipopolysaccharide (LPS, 100 ng/ml) or both for 24 h. Subsequent immunoblots for β-actin (ACTB) are shown for equal protein loading.

To explore the mechanisms underlying reduced IBA1/AIF1 expression in *Irf8*^-/-^ microglia *in vivo*, we examined IBA1/AIF1 expression in *Irf8*^+/+^ and *Irf8*^-/-^ microglia *in vitro*. In agreement with our *in vivo* results and another study [16], *Irf8*^-/-^ microglia expressed far less IBA1/AIF1 protein than *Irf8*^+/+^ microglia in the control cultures (Figure 7B). The qPCR results also demonstrated that the basal levels of *AIF1/IBA1* mRNA in *Irf8*^-/-^ microglia were approximately five-fold lower than those in *Irf8*^+/+^ microglia (Figure 7A). However, both AIF1/IBA1 mRNA and protein were upregulated by IFNγ even in the absence of IRF8. Interestingly, this IFNγ-mediated induction of AIF1/IBA1 was significantly inhibited by simultaneous stimulation with LPS, particularly in *Irf8*^-/-^ microglia (Figure 7). 

### Altered cytokine induction in IRF8-deficient microglia in response to LPS and IFNγ

**Figure 8:**
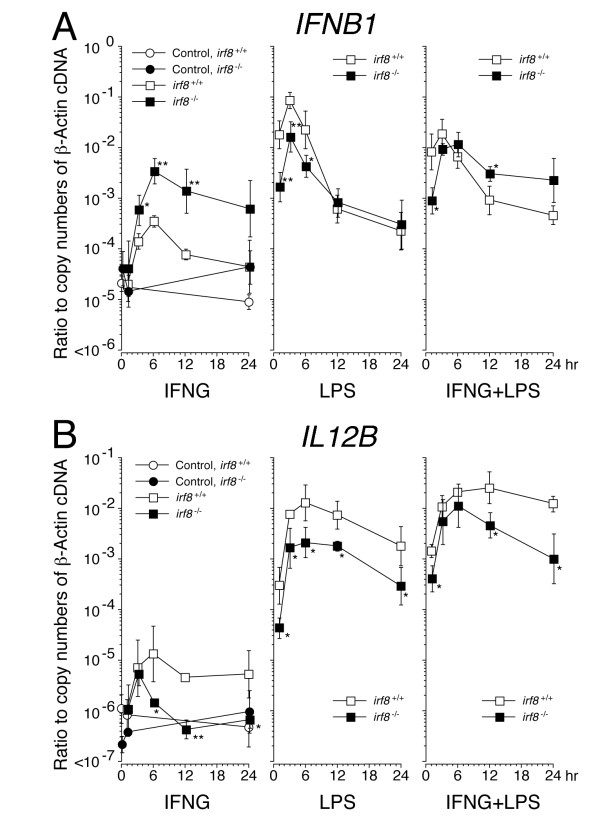
**Kinetics of *IFNB1 *(A) and *IL12B *(B) mRNA levels in *Irf8***^**+/+ **^**and *Irf8***^**-/- **^**microglia after the exposure to IFNγ and lipopolysaccharide.** Purified microglia were incubated in medium alone (Control, closed and open circle in the left panels) or in the presence of IFNγ (IFNG, 100 ng/ml), lipopolysaccharide (LPS, 100 ng/ml) or both (IFNG + LPS) for 24 h. *IFNB1* and *IL12B* mRNA at the indicated time points were quantified by qPCR and are plotted as ratios to copy numbers of β-actin cDNA on a logarithmic scale. Data were calculated from at least three independent experiments in each genotype. At time 0, data from controls are only shown. **P < 0.01 and *P < 0.05, in a comparison of the two genotypes at the indicated time points.

In BM-derived myeloid cells, IRF8 is critically involved in transcriptional regulation of various cytokines which are essential for initiation and modulation of the adaptive immune response [14,25,26]. Among those cytokines, we asked whether IRF8 regulates the transcriptional induction of *Interferon-β1* (*IFNB1*) and *Interleukin-12b* (*IL12B*) in response to LPS and IFNγ in microglia in the same manner as reported in preceding studies using BM-derived myeloid cells. As shown in Figure 8A, quantitative and kinetic analysis of *IFNB1* mRNA revealed that IRF8 contributes to the transcriptional regulation of *IFNB1* in microglia in at least two ways; First, IRF8 positively regulates acute transcriptional induction of *IFNB1* in response to LPS, which is a distinctive mode of induction in myeloid cells [27]. In the absence of IRF8, however, this rapid induction of *IFNB1* still occurs at approximately ten-fold lower levels. Second, IRF8 represses IFNγ-mediated induction of *IFNB1*. The qPCR results of *IL12B* mRNA demonstrated that IRF8 acted as a strong transcriptional enhancer in both IFNγ- and LPS-mediated transcriptional induction of *IL12B* in microglia (Figure 8B). 

### IRF8-deficient microglia demonstrated a delayed response to tissue damage and a defect in clearing damaged tissues in the cuprizone demyelination model

**Figure 9:**
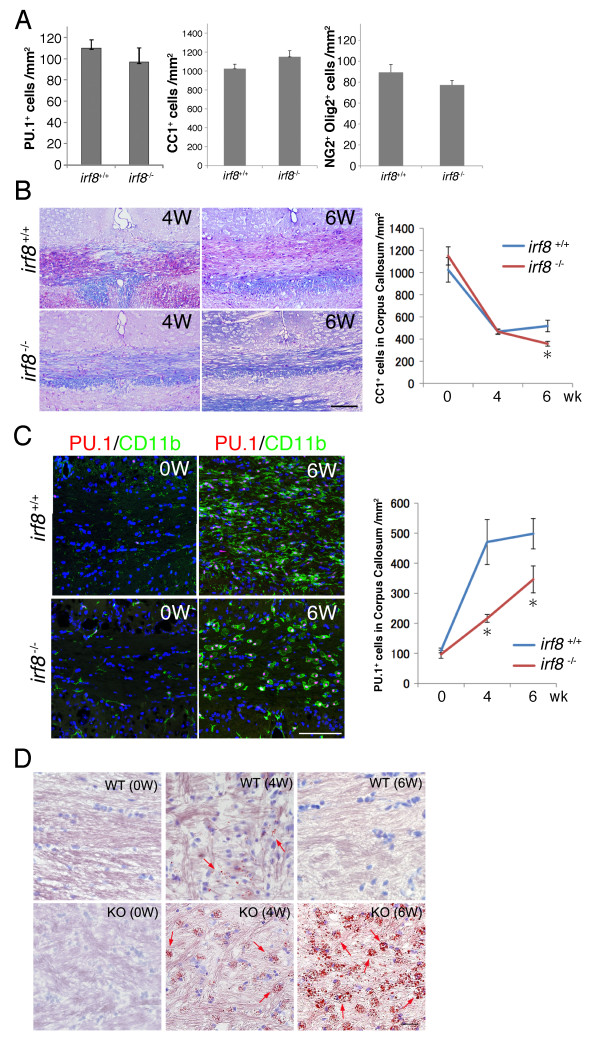
**A defective phenotype of IRF8-deficient microglia *in vivo *in the cuprizone-induced demyelination model.** (**A)** There was no quantitative difference in corpus callosal microglia, myelinating oligodendrocytes, and NG2-positive oligodendroglial precursor cells between *Irf8*^+/+^ and *Irf8*^-/-^ mice before cuprizone feeding. Microglia and myelinating oligodendrocytes were identified by immunoreactivity for nuclear PU.1 and for the cytosolic CC1 epitope, respectively. (**B)** Cuprizone-mediated demyelination in the medial corpus callosum in *Irf8*^+/+^ and *Irf8*^-/-^ mice. Representative LFB-PAS-stained paraffin sections of the medial corpus callosi of *Irf8*^+/+^ and *Irf8*^-/-^ mice at 4 and 6 weeks of cuprizone diet (left panel). Scale bar: 100 μm. Reduction of myelinating oligodendrocytes in the corpus callosum. CC1^+^ oligodendrocytes per unit area of 6 μm-thick coronal sections were counted. At least three mice were used for each data point (right graph). (**C)** Microglial accumulation in the corpus callosum was delayed in *Irf8*^-/-^ mice compared with *Irf8*^+/+^ mice. CD11b^+^ microglia with PU.1^+^ nuclei were quantified in 6 μm-thick coronal sections as in **B** (right graph). Note that the abnormal oval cell shape of activated *Irf8*^-/-^ microglia at 6 weeks of cuprizone feeding. Scale bar: 100 μm. (**D)** Oil red O plus hematoxylin staining of the corpus callosum of *Irf8*^+/+^ and *Irf8*^-/-^ mice at 0, 4 and 6 weeks of cuprizone feeding. Lipid-rich myelin debris visualized by Oil red O staining (red arrows) rapidly accumulated in the corpus callosum of *Irf8*^-/-^ mice, as demyelination progressed. Scale bar: 25 μm. Data are presented as mean ± standard error. *P < 0.05 by Mann-Whitney *U* test.

We finally examined whether *Irf8*^-/-^ microglia are defective in their functions *in vivo*. As we consider microglia as a myeloid population distinct from BM-derived myeloid cells, it was essential to evaluate their functions with minimum contributions of BM-derived myeloid cells from the systemic circulation. Cuprizone-induced demyelination in mice is an established rodent model in which cuprizone in the diet selectively kills myelinating oligodendrocytes particularly in the corpus callosum. Importantly, the blood brain barrier remains intact during this intoxication [28], and contributions of BM-derived MPs and T lymphocytes from the circulation are quantitatively small [29,30]. We first confirmed that numbers of microglia, myelinating oligodendrocytes, and their precursors that are recognized by surface expression of NG2 chondroitin sulfate proteoglycan (CSPG4) were not altered quantitatively in the corpus callosum of *Irf8*^-/-^ mice before cuprizone intoxication (Figure 9A). Six week-feeding with 0.25% (w/w) cuprizone induced demyelination in the corpus callosum in both *Irf8*^+/+^ and *Irf8*^-/-^ mice, although more myelin remained in the corpus callosum of *Irf8*^-/-^ mice than in *Irf8*^+/+^ mice. However, surviving myelinating oligodendrocytes labeled with CC1 monoclonal antibody were reduced similarly in both *Irf8*^+/+^ and *Irf8*^-/-^ mice during cuprizone feeding, indicating the same cytotoxicity of cuprizone to *Irf8*^+/+^ and *Irf8*^-/-^ oligodendrocytes (Figure 9B). Nevertheless, we found delayed accumulation of microglia double labeled with CD11b and PU.1/SFPI1 in the demyelinated corpus callosum of *Irf8*^-/-^ mice. Interestingly, the activated *Irf8*^-/-^ microglia had characteristic CD11b-positive oval cell soma with a clear margin, whereas activated *Irf8*^+/+^ microglia extended CD11b-positive cell soma diffusely, which made it difficult to distinguish each *Irf8*^+/+^ microglia by single label staining for CD11b (Figure 9C). More notably, oil red O staining which visualized myelin lipids revealed accumulation of myelin debris in *Irf8*^-/-^ mice, indicating defective scavenging activity of *Irf8*^-/-^ microglia (Figure 9D). 

## Discussion

The first finding in this study is that, even in the absence of IRF8, microglia can develop and colonize the CNS. This contrasts clearly to inefficient development of BM-derived MPs and accumulation of myeloid progenitor cells in *Irf8*^-/-^ mice [11], and indicates that IRF8-mediated transcriptional regulation is not necessary for development of microglia in the primitive hematopoiesis and for their migration into the embryonic CNS. On the other hand, loss of PU.1/SFPI1 blocks MP development as early as the primitive hematopoiesis in the yolk sac, resulting in lack of tissue residential MPs including microglia, liver Kupffer cells, and lung alveolar macrophages [31]. IRF8 is known to associate with PU.1/SFPI1 to activate transcription of some genes by interacting with the composite Ets/IRF-cis elements in the promoter region [32,33]. However, our result indicates that PU.1/SFPI1 does not require IRF8 to exert its critical roles in development of microglia. After migration into the CNS, a large proportion of the adult microglial cell population arises during the first two postnatal weeks by an intense *in situ* proliferation in proportion to the growth of the CNS tissues [34]. Based on the observations on the mutant mice lacking CSF1R [7,35] or one of its ligands, M-CSF [36,37], this perinatal expansion of the microglial population size is largely dependent on mitotic signals mediated by the M-CSF receptor CSF1R/CD115. We can conclude from our *in vivo* and *in vitro* experiments that, even in the absence of IRF8, microglia can respond to M-CSF and proliferate.

However, colonized IRF8-deficent microglia are not phenotypically and functionally equivalent to wild-type microglia. An obvious difference is that the processes of *Irf8*^-/-^ microglia in the steady state are less extended into the tissues compared to those of *Irf8*^+/+^ microglia. Recent two-photon *in vivo* or *ex vivo* imaging has revealed that, although ramified microglia in the steady state have been referred as ‘resting’ microglia, their processes are highly dynamic to probe changes actively in their microenvironment [38] and to monitor neuronal synaptic activities [39]. If microglia detect tissue damage, their processes can quickly converge to form a barrier to contain a damaged area from the surrounding healthy tissue [40,41]. Our observation suggests that *Irf8*^-/-^ microglia may be less capable of sensing tissue damage due to their less extended processes. IRF8-deficent microglia will provide a unique model for further understanding of process dynamics and sensing capability of microglia.

Reduced expression of AIF1/IBA1 is also a prominent feature of *Irf8*^-/-^ microglia [16]. As far as we could examine, however, AIF1/IBA1 is the only microglial marker affected by loss of IRF8, indicating again that a major part of microglial development occurs normally in the absence of IRF8. Our analysis of *AIF1/IBA1* mRNA and protein levels demonstrated that IRF8 is required to maintain the constitutive expression of AIF1/IBA1 in microglia, whereas AIF1/IBA1 is still inducible in *Irf8*^-/-^ microglia by IFNγ. AIF1/IBA1, a 17-kDa protein bearing two EF-hand Ca^2+^ binding motifs, is known to contribute to the plasma membrane and cytoskeleton dynamics tightly linked to motility and phagocytosis of microglia [42]. It is thereby reasonable to speculate that impaired process formation in *Irf8*^-/-^ microglia might be a consequence of reduced AIF1/IBA1 expression. Despite the report that AIF1/IBA1 is involved in the formation of phagocytic cups in a microglial cell line [42], however, engulfment of zymosan particles occurred in *Irf8*^-/-^ microglia in the same time frame as that in *Irf8*^+/+^ microglia, indicating that the molecular mechanisms underlying a series of microglial responses from chemotaxis to phagocytosis [43] are not impaired by the reduction of AIF1/IBA1 protein. A hypothetical explanation for this discrepancy would be that the trace amount of AIF1/IBA1 protein in *Irf8*^-/-^ microglia might be sufficient for the phagocytic responses. Alternatively, AIF1L/IBA2, a homolog of AIF1/IBA1, might compensate the reduced AIF1/IBA1 [44]. Basal expression of *AIF1/IBA1* mRNA in *Irf8*^-/-^ microglia, though it is five-fold reduced, is likely to be attributable to PU.1/SFPI1 that was expressed at the levels similar to those in *Irf8*^+/+^ microglia, because PU.1/SFPI1 has been shown to bind to the promoter region of the *aif1/iba1* gene and act as a transcriptional activator [45]. Further studies will be necessary to determine whether IRF8 enhances expression of AIF1/IBA1 directly or indirectly in cooperation with PU.1/SFPI1 in microglia.

In contrast to normal zymosan engulfment, the maximum phagocytic capacity of *Irf8*^-/-^ microglia is reduced as determined by the amount of zymosan particles engulfed in each microglia. After engulfment, phagosomes containing internalized materials such as bacteria and apoptotic cell corpse undergo a maturation process along with sequential changes in integral membrane proteins and progressive acidification, and eventually fuse with lysosome structures to form phagolysosomes, leading to degradation of the contents [46,47]. IRF8-mediated transcriptional regulation might be involved in this highly organized maturation process of phagosomes, or cell structural changes associated with the phagocytic activities. Defective scavenging activity of *Irf8*^-/-^ microglia was also observed in our *in vivo* experiment using the cuprizone-induced demyelination model. More myelin debris rich in lipids remained in the corpus callosum of *Irf8*^-/-^ mice. However, this *in vivo* result may be attributable not only to reduced phagocytic capacity of *Irf8*^-/-^ microglia, but also to delayed accumulation of *Irf8*^-/-^ microglia in the demyelinating lesions.

After development of the CNS, microglia continue to cycle slowly [48], and are capable of accelerated proliferation particularly in response to tissue damages [30,49,50]. Although the molecular basis of this proliferative response associated with microglial activation might differ depending on the pathological conditions, CSF1R/CD115-mediated signaling is principally involved in microglial proliferation in some types of CNS lesions such as facial motoneuron death after axotomy [51,52]. Microglia also proliferate *in vitro* in response to various cytokines including M-CSF, GM-CSF, interleukins-3, -4, and -5 [53-57]. An interesting finding in our *in vitro* experiments was that *Irf8*^-/-^ microglia demonstrated significantly reduced proliferation in mixed glial cultures, whereas exogenous M-CSF restored proliferation of *Irf8*^-/-^ microglia at a rate comparable to that of *Irf8*^+/+^ microglia. Exogenous M-CSF also enhanced proliferation of purified CD11b^+^*Irf8*^-/-^ microglia, but to a lesser extent compared with purified CD11b^+^*Irf8*^+/+^ microglia, suggesting that IRF8-dependent transcription in microglia is required for their normal proliferative response to M-CSF at a physiological concentration. Since a previous *in vitro* study using M-CSF-deficient mice indicated that M-CSF derived from astroglia principally contributes to microglial proliferation in mixed glial cultures [58], however, IRF8-dependent mechanisms in astroglia might be involved in secretion of colony stimulating factors including M-CSF from astroglia. In addition, the reduced proliferative response of *Irf8*^-/-^ microglia in the mixed glial cultures could at least partly account for delayed accumulation of *Irf8*^-/-^ microglia in the cuprizone-induced demyelinated lesions.

GM-CSF is known to be a potent mitogen for wild-type microglia *in vitro* as well [53,56,59]. In mixed glial cultures, *Irf8*^-/-^ microglia demonstrated a hyperproliferative response to GM-CSF compared with *Irf8*^+/+^ microglia, which is quite likely to be identical to that observed with *Irf8*^-/-^ BM-derived myeloid progenitor cells [22]. However, our results indicated that this hyperproliferative response of *Irf8*^-/-^ microglia requires the presence of other glial cells, because exogenous GM-CSF failed to enhance proliferation of *Irf8*^-/-^ microglia after isolation from other glial cells. A straightforward explanation for these findings is that GM-CSF acts indirectly on microglia through GM-CSF-mediated production of other mitogenic factor(s) from astroglia, and that IRF8 is involved in the proliferative response of microglia to these factors. Since some prior studies have pointed out that, in the absence of astroglia, GM-CSF failed to induce proliferation of microglia particularly from adult animals [60,61], it is also conceivable that GM-CSF-induced proliferation of microglia is dependent on their maturational and activation stages. Further studies will determine whether microglia share the same IRF8-dependent intracellular signaling pathways leading to the mitotic response to GM-CSF as BM-derived myeloid progenitor cells [62,63]. GM-CSF also induces a phenotypic skew of microglia towards DC-like cells, but not towards granulocytes [60]. We observed that this phenotypic skew was more apparent in *Irf8*^-/-^ microglia than in *Irf8*^+/+^ microglia. Given that IRF8 positively regulates development of diverse BM-derived DC subsets in a subset-selective manner [64], roles for IRF8 in microglial differentiation towards a DC phenotype in the presence of GM-CSF might be distinct from those in BM-derived common DC progenitors.

We also confirmed that *Irf8*^-/-^ microglia have the same abnormality in the transcriptional induction of *IFNB1* and *IL12B* as that reported in *Irf8*^-/-^ BM-derived myeloid lineage cells. Our quantitative and kinetic analysis of *IFNB1* mRNA clearly demonstrated that IRF8 positively regulates LPS-mediated (Toll-like receptor 4-mediated) acute induction of *IFNB1* mRNA, whereas it suppresses IFNγ-mediated delayed transcriptional induction in microglia. Recently, Li *et al.* reported that, using human monocytes, IRF8 positively regulates rapid and robust induction of IFNβ in cooperation with IRF3 and PU.1, and that this mode of induction is characteristic to monocytes [27]. Our result is in good agreement with their findings, indicating that microglia utilize the same transcriptional machinery in the *IFNB1* promoter as BM-derived monocytes. IRF8 is also an essential transcription factor for induction of IL12 p40 subunit in BM-derived MPs [14,26]. Although our result confirmed the same positive regulatory role for IRF8 in microglia, we also noticed that LPS-mediated *IL12B* mRNA induction still occurred in the absence of IRF8 at ten-fold lower levels, suggesting the presence of a compensatory mechanism in microglia.

Our study provides further evidence for differences between microglia and BM-derived MPs with respect to the dependence of their development and physiological phenotypes on IRF8-mediated transcriptional regulation. Detailed gene expression analysis of *Irf8*^+/+^ and *Irf8*^-/-^ microglia, and *Irf8*^+/+^ and *Irf8*^-/-^ BM-derived MPs will be necessary to clarify the downstream events leading to the difference in gene expression profiles between microglia and BM-derived MPs [65,66].

## Conclusions

In conclusion, our comprehensive analyses of IRF8-deficient microglia demonstrate that IRF8 is a critical transcription factor for a broad range of microglial functions, some of which are unique in microglia. In line with our findings, recent studies have revealed direct association of IRF8 with human disease conditions [67,68]. This study warrants future studies to enhance understanding of critical contributions of IRF8-mediated transcriptional regulation to physiological and pathological roles of microglia.

## Abbreviations

BM: Bone marrow; BMDM: BM-derived microglia; BSA: Bovine serum albumin; CSF1R: Colony stimulating factor 1 receptor; DAPI: 4,6-diamidio-2-phenylindole; DC: Dendritic cell; DMEM: Dulbecco’s modified Eagle’s medium; EdU: 5-ethynyl-2′-deoxyuridine; G-CSF: Granulocyte colony stimulating factor; GM-CSF: Granulocyte-macrophage colony stimulating factor; HBSS: Hanks’ balanced salt solution; ICSBP: interferon consensus sequence binding protein; IFNB1: Interferon-β1; IL12B: Interleukin-12b; IRF: Interferon regulatory factor; LFB: Luxol fast blue; LPS: Lipopolysaccharide; MACS: Magnetic cell sorting; M-CSF: Macrophage colony stimulating factor; MFI: Mean fluorescent intensity; MP: Mononuclear phagocyte; PAS: Periodic acid Schiff; qPCR: real-time PCR.

## Competing interests

The authors declare that they have no competing interests.

## Authors’ contributions

MH contributed to experimental design, carried out most of the *in vitro* experiments, analyzed the data, and helped to write the manuscript. KW contributed to experimental design, carried out most of the *in vivo* experiments, analyzed the data, and helped to write the manuscript. AI performed a part of the biochemical experiments, and helped with genotyping and colony maintenance of the experimental animals. KK performed an initial analysis of IRF8-deficient mice. DP participated in data interpretation and critical reading of the manuscript. KO provided the experimental animals and anti-IRF8 antibody, and participated in critical reading of the manuscript. TI conceived the study, contributed to experimental design, and wrote the manuscript. All authors read and approved the final manuscript.
